# Prevalence and Factors Associated with Postpartum Depression Among Mothers Attending Public Health Centers of Addis Ababa, Ethiopia, 2016

**DOI:** 10.2174/1745017901814010196

**Published:** 2018-08-31

**Authors:** Addishiwet Fantahun, Amsale Cherie, Leul Deribe

**Affiliations:** Department of Nursing and Midwifery, Collage of Health Science, Addis Ababa University, Addis Ababa, Ethiopia

**Keywords:** Postpartum depression, Maternal mental health, Postpartum period, (EPDS), Addis ababa, Public health

## Abstract

**Objectives:**

Postpartum Depression (PPD) is a serious public health problem that leads to high maternal morbidity and mortality, enormously affecting the infant, family and society. Thus, the aim of this study was to assess the prevalence and factors associated with postpartum depression among postpartum mothers attending public health centers in Addis Ababa, Ethiopia, 2016.

**Methods:**

Facility-based cross-sectional study was conducted from March 2016-April 2016 among 633 postpartum women. Four sub cities were identified through simple random sampling technique among 10 sub cities in Addis Ababa, Ethiopia. Furthermore, the study participants were determined by systematic random sampling after 10 health centers were selected by lottery method and the number of participants in each health center was proportionally allocated. In order to determine postpartum depression, participants were rated using the Edinburgh Postnatal Depression Scale (EPDS) and the findings were analyzed using bivariate and multivariate logistic regression. P-value less than 0.05 with 95% confidence interval was used to state the association.

**Results:**

The study revealed prevalence of postpartum depression among mothers was 23.3%. Moreover, women who were unmarried, had unplanned pregnancy, delivered without presence of any relatives in health institutions, had previous history of child health, had history of substance use and had low income were found to more often display postpartum depression.

**Conclusion:**

For optimal maternal health care provision in regards to postpartum depression, integration of mental health service in addition to inter sectoral collaboration of women’s affair with health institutions is crucial.

## 1 INTRODUCTION

Postpartum Depression (PPD) involves various groups of depressive symptoms and syndromes that take place during the first year following birth [1]. It is recognized as risk period for severe mood disorder that comprises provisional blue, major depression and debilitating psychotic depression [2]. Several sign and symptoms that define PPD are low self-worth and interest, tiredness, sadness, disturbed sleep and appetite [3], problem in concentrating and making decision, feeling of unworthy to live, having negative thought about the baby, feeling of guilt and shame [4].

Worldwide, statistics shows that 450 million people are seriously affected by neurological and mental illness ranking depression as the fourth principal cause for disability and premature deaths and by the year 2020, depression is predicted to be the second leading cause of disability [5]. Irrespective of economic status, race or ethnic groups’ depression tends to occur twice more in women than men [2].

Globally, about 10% of pregnant women and 13% of women who just gave birth are suffering from mental health problems. It is higher in developing countries where 15.5% develop mental illness during pregnancy and 19.8% after childbirth [3] continuing to affect the welfare of mothers, their babies, partners and family members [6].

A number of researches have been done in developed countries while limited facts from developing countries including Ethiopia are found [7, 8]. PPD has been studied in more than 90% of high income countries (HICs) compared with just 10% of low and middle-income countries (9). According to the Ethiopian national mental health strategy in the year 2012/13 - 2015/16 by Federal Democratic Republic of Ethiopia Ministry of Health (FMOH) mental illness was the leading non-communicable disorder in terms of burden. Indeed, in a predominantly rural area of Ethiopia, mental illness comprised 11% of the total burden of disease including schizophrenia and depression among the top ten of the most burdensome illness. The prevalence for general depression was 5.0% and more than one in ten pregnant women and one in 20 postnatal women in Ethiopia suffer from undetected depression [10].

As provision of care will vary depending on the socio-demographic and cultural factors, it is difficult to establish conclusion on prevalence and associated risk factors of PPD. Despite its massive effects especially in low and lower income countries, women and clinicians inadequately understand it. Even though multiple studies have been conducted in Ethiopia concerning postpartum cares, the focus has always been on PNC or family planning services whereas the prevalence and associated factors of PPD gained little attention. As a result, it is important to get an insight and plan for the implementation strategies to prevent and identify PPD early at the postpartum period. Therefore, the present study aims at assessing the prevalence and associated factors of postpartum depression among mothers attending public health centers in Addis Ababa, Ethiopia.

## 2 MATERIALS AND METHODS

### 2.1 Study Area

The study was conducted in Addis Ababa city, the capital city of Ethiopia and the seat of the African Union. It has a total population of 3,048,631. Addis Ababa has 11 government owned hospitals, 90 health centers, 31 private hospitals and 700 different level private clinics. Each sub-city has more than one health centers. Health centers are easily accessible for the community and maternal health services provided freely. They are supposed to provide a package comprising both preventive public health and essential curative services. The health centers have a capacity of 10 beds, and are open for 24 hours in a day to provide curative health, emergency service and maternal and child health services. Health centers are usually staffed by health officers or/and a doctor, clinical nurses, midwives, and other health personnel including administrative staff [8].

### 2.2 Study Design and Study Setting

This facility based cross sectional study was conducted in health centers of Addis Ababa, Ethiopia from March –April 2016.

### 2.3 Sample Size Determination and Sampling Technique

The sample size was calculated based on single population proportion formula with 95% confidence level (CI), 5% margin of error (d), and 50% proportion (p) of prevalence of postpartum depression. By adding 10% non-response rate and a design effect of 1.5, a total sample size of 633 was taken.

Multistage sampling technique was employed to select the respondents of the study. First out of ten sub-cities found in Addis Ababa city government, four sub cities namely Lideta, Nifasilik lafto, Kirkos and Gulele were selected using simple random sampling method. Secondly, out of 33 health centers found in the selected four sub-cities, 10 (two from Lideta and Nifasilik lafto each and three from Gulele and Kirkos each) Health centers were selected by a lottery method.

The number of women included in the study from the selected health centers was determined using proportion to size allocation technique based on previous three-month data of selected health centers. To identify the interval, average number of women expected per day in each health center was divided by number of women to be interviewed per day from respective health centers. The first woman was selected by lottery method and then every other two women visiting the health center were nominated for the study.

All women who came for postnatal care and vaccination service within 6 weeks after delivery in selected health centers during data collection period and consented to participate in the study were included while women who were seriously sick, unable to respond to the questions and those who were refusing to participate in the study were excluded.

### 2.4 Data Collection

A structured interviewer-administered questionnaire was adopted from previously published literatures [9, 11-17]. The questionnaire was designed in English and translated into the local language (Amharic) by experts in the field and subsequently translated back into English by a different expert to check for consistency. The Amharic version of the questionnaire was used for date collection.

Pretest was done with 10% of the sample size in a health center other than the study settings. Five diploma nurses who were not employees of the selected health centers collected data. The data collectors and supervisor were trained for one day on the objective, data collection techniques, maintaining confidentiality, data quality, and techniques of interview. One nurse and the principal investigator closely supervised the data collection process, while data consistency and completeness were checked in daily basis.

### 2.5 Measurement

The dependent variable was postpartum depression while the independent variables were Socio-demographic characteristics, Obstetrics factors, Substance use and Social support. Postpartum depression was measured by Edinburg Postnatal Depression Scale (EPDS) which indicates how the mother has felt during the previous 7 days at a cutoff point >13. Mothers who scored above the cutoff point were considered to have postpartum depression. The EPDS generated sensitivity and specificity of 78.9% and 75.3% respectively [18].

The collected data were checked for completeness and were entered into EpiData 3.5, then, the analysis was made with Statistical Package for Social Science (SPSS) versions 23. Descriptive summaries were used to describe the study variables. Variables with a p-value <0.2 in bivariate analysis were entered into the multivariate logistic regression to control for possible confounding variables. The p-values <0.05 or 95% CIs not including 1.0 were considered to indicate statistical significance.

### 2.6 Ethical Considerations

Ethical approval was obtained from the research ethical committee of Addis Ababa university department of nursing and midwifery. Written consent was attained from Addis Ababa administrative health bureau and formal letter was written from the health bureau to the selected sub cities. After Permission was sought from the responsible bodies of the health centers, written consent was obtained from each participant after the investigator had explained the nature, purpose and procedure of the study.

## 3 RESULTS

### 3.1 Socio-demographic Characteristics

Of the total 633 sampled post-partum women, 618 (97.6%) participants responded fully to all the questions. The mean age of respondents were 28.05 with standard deviation of 5.0 and a median age of 28 years. From the total study subjects, 526 (85.1%) were married. Majority of the participants 502 (81.2%), attended formal education whereas 281 (45.5%) of the respondents were unemployed. Of the 210 (34.0%) participants reported having low family income, 170 (27.5%) confirmed to earn an average of less than 445 Ethiopian birr per month (Table **1**).

### 3.2 Obstetric Characteristics

Of all the respondents, 228 (36.9%) identified the recent pregnancy as their first and 179 (29.0%) reported that the pregnancies were unplanned while 21 (3.4%) had stressful life events during recent pregnancies. One hundred sixty four (26.5%) experienced illnesses during pregnancy, 131 (21.2%) delivered through caesarean section, 40 (6.5%) had experienced death of a child and 104 (16.8%) hospitalization of their babies in their lifetime. Sex of the last baby was found to have a female to male distribution of 50.3% to 49.7% respectively and 136 (22%) of the respondents were not satisfied with the sex of their infants (Table **2**).

### 3.3 Substance use Among Postpartum Women

Ninety (14.6%) and 16 (2.6%) of the study participants claimed they had used substance before and during recent pregnancy, respectively. In addition, the substance mostly used was determined to be alcohol (areke, tela, tej, beer and wayn).

### 3.4 Social Support Among Postpartum Women

Even though the majority of participants were satisfied with their marriage, 113(18.8%) of them described their relationship with their husband as unsatisfactory and 104 (16.8%) of the respondents lacked assistance from their husbands. It has also been found that 58 (9.4%) of respondent’s relatives were not present at the health facilities during labor and 178 (28.8%) reported as being unhappy with the relationship they had with their mother in law (Table **3**).

### 3.5 Prevalence of Postpartum Depression

**Fig. (1):**
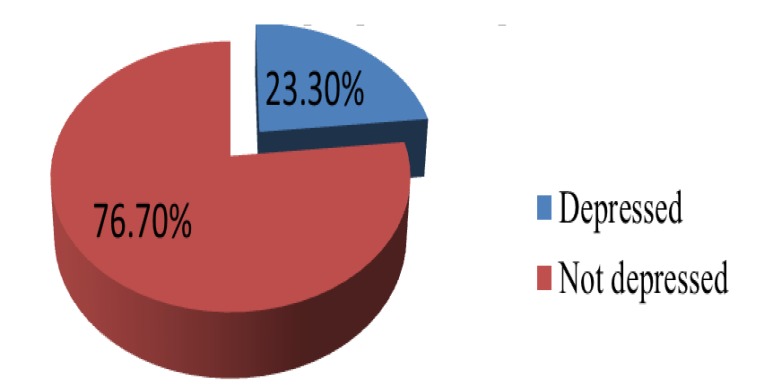
Prevalence of postpartum depression among postpartum women’s, from health centers of four sub-cities of Addis Ababa city Administration, Ethiopia, (N= 618).

From all the respondents, 144 (23.3%) had postpartum depression (Fig. **1**) and 69 (11.2%) reported they were not “able to laugh and see funny side of things”. For forty-eight (7.8%) of the participants it was “difficult to look forward with enjoyment to things” and 58 (9.4%) were frequently blaming themselves unnecessarily. As nearly one sixth of the study participants were anxious or worried for no good reason, 30 (4.9%) reported worrying about scaring or panicking for no good reason and 25 (4.0%) stated they were not able to cope up with things at all. Twenty-six (4.2%) of the study participants had difficultly to sleep and 21 (3.4%) respondents reported that mostly they “felt sad or miserable”. In addition, 18 (2.9%) were unhappy and have been crying most of the time whereas two (0.35%) had a thought of harming themselves (Table **4**).

### 3.6 Factors Associated with Postpartum Depression

Eleven variables showed association with postpartum depression at the bivariate level and were entered into multivariate analysis. (Table **5**). Of this variables six were significant in the multivariate analyses (AOR; 95% CI): being unmarried (7.7; 3.0-19.5), previous history of child death (2.7; 1.6-4.4), income difficulty (2.9; 1.3-6.4), delivered without the presence of any relatives (5.5; 2.6-11.6), history of substance use (2.6; 1.4-4.8), planned pregnancy (0.2; 0.1-0.3).

## 4 DISCUSSION

The current study assessed the magnitude and factors that has an association with post-partum depression in selected health centers of Addis Ababa. The prevalence of PPD among mothers who came for postnatal and vaccination services in sampled health centers was 23.3%. This result implied that a significant proportion of women were experiencing PPD, indicating that maternal mental health problem is a substantial concern for which services are urgently needed. This result was comparable to studies conducted in China, a city of Poland and Lahore, Pakistan, where PPD was presented among 27% [10], 23% [16] and 25% [19] of women given birth, respectively. On the other hand, this figure was substantially higher when compared to other similar studies done in Japan [20], Canada [21], Qatar [22], Turkey [23] and Sudan [24] where the corresponding proportion of women displaying PPD was 8%, 9%, 19%, 15% and 9%, respectively. However, this figure is less compared to other similar studies done in the Ethiopian regions, Amhara (33%) and Oromia, Bale zone (32%) [25, 26]. Likewise, studies from Iran [27], South Africa [14] and Uganda [28] also reported a high prevalence of PPD. The difference between the present study and previous reports might be due to usage of different investigation tools, different sample sizes various assessment periods, different methods in the assessment of PPD and economic status of the regions investigated.

PPD was significantly higher among unmarried than those who were married. This finding is in line with studies carried out in United States [11, 29], Uganda [30] Jamaica [31] and Amhara region in Ethiopia [25]. This might be due to the fact that, during the period of pregnancy and delivery unmarried women were handling the situation alone and did not have the support they needed from a partner.

Furthermore, PPD was significantly higher among participants who had low income compared to those who did not. This was similar to other studies conducted in Saudi Arabia [32], Qatar [22], Korea [33], western Iran [27], south India [34], and Japan [20]. This might be because of overstressing to provide their children with everything they feel they require despite financial constraints and may in turn be related to having experienced stressful life events related to money during pregnancy or previous in life. More likely so, another significant association found in this study was between depression and previous history of child death in which PPD was significantly higher among participants who lost a baby at least once than among those did not. This result is consistent with the 2012 Communicable Disease Control (CDC) report on “ depression among women of reproductive age” [15] and another study, which was Conducted Among reproductive age groups in South East Ethiopia, Oromia region, Goba and Robe town of Bale zone [26]. The association between a previous history of child death and PPD might be because serious negative life events are most influential on individuals’ mental status; it is also possible that the association could be explained by the pregnant women’s fear of losing their newborn as well.

An increased odd of PPD among women who did not have a planned pregnancy was also observed. Most, but not all previous studies, *e.g.* investigations carried out in Qatar [22], Turkey [23], Iran [35] and northwestern Brazil [36] identified unplanned pregnancy to be associated with PPD, whereas another report from Iran did not find evidence for this relationship [37]. The difference might be due to economical variation between the different regions investigated. A study which was conducted among pregnant mothers who follow antenatal service in Addis Ababa, Ethiopia also highlighted that unplanned pregnancy had a contribution for antenatal depression [38].This indicates that unplanned pregnancy is a factor for both antenatal depression and PPD. Although, the family planning service coverage in Ethiopia has increased, unplanned pregnancy is still common implying that there is still a gap in utilization of the service.

The other variable that was significantly associated with PPD was history of substance use (alcohols (‘tela’, ‘teji’, beer and Wayne), ‘chat’ (an evergreen plant which has sympathomimetic effect when gradually chewed); cigarettes and water pipe smoking (shisha). This might be because women have lack of awareness about the effect of drinking before and during pregnancy especially homemade drinks. This is indicative of the fact that women should receive adequate information regarding the effect of alcohol drinks on pregnancy whether commercial or homemade. The finding was similar to other studies where postpartum depression was assessed in women between 3 and 52 weeks postpartum [39]. In other cross sectional study, among 43,093 adult women, 12.4% of the respondents were exposed to major depression in which 35.4% were nicotine dependent [29].

In addition, PPD was significantly higher among women who gave birth without the presence of any relatives in the health institutions and who did not get social support during labor and delivery. This result was consistent with a study conducted in Bydgoszcz city of Poland [16]. The finding identified that the presence of any relatives, especially husbands during labor and delivery was helpful for emotional support other than giving social support.

## CONCLUSION

This study found that 23.3% of respondents had PPD, which is significantly a high value. It also identifies the presumed risk factors; Socio-demographic factors like marital status and low economic status were associated with PPD. Similarly, women who had previous history of child death and substance use had a higher probability of being depressed at the postpartum period. Unplanned pregnancy and childbirth without the presence of any relatives were also among the factors which were identified. Incorporating mental health service with existing or present maternal health care service in addition with inter sector collaboration between women’s affair and health institutions is recommended to prevent PPD.

## Figures and Tables

**Table 1:** Socio-Demographic characteristics among women in postpartum period, in health centers of four sub-cities of Addis Ababa city Administration, Ethiopia, (N=618).

**Characteristics**	**Frequency**	**Percent**
**Age in years**		
15-24	138	22.3
25-34	410	66.3
>35	70	11.3
**Marital status **		
Married	526	85.1
Unmarried	92	14.9
**Attended school **		
Yes	502	81.2
No	116	18.8
**Highest level of education **		
Primary school	124	24.0
Secondary school	110	21.3
Technical or vocational	60	11.6
Diploma	115	22.2
Degree and above	108	20.9
**Occupational status **		
Employed	337	54.5
Unemployed	281	45.5
**Difficult with income **		
Yes	210	34.0
No	408	66.0
**Monthly average income **		
<445	170	27.5
446-1200	64	10.4
1201-2500	97	15.7
2501-3500	96	15.5
>3501	191	30.9

**Table 2:** Participants Obstetrics and clinical characteristics in postpartum period, from health centers of four sub-cities of Addis Ababa city Administration, Ethiopia, (N= 618).

**Characteristics**	**Frequency**	**Percent**
**Number of pregnancy **		
1	228	36.9
2-3	311	50.3
>4	79	12.8
**Planed pregnancy **		
Yes	439	71.0
No	179	29.0
**Sex of last baby **		
Male	307	49.7
Female	311	50.3
**Desired sex of the baby **		
Desired	207	33.5
Undesired	136	22.0
I don’t mind	275	44.5
**Mode of delivery**		
Vaginal	416	67.3
Cesarean section	131	21.2
Instrumental delivery	71	11.5
**Illness during pregnancy **		
Yes	164	26.5
No	454	73.5
**Experience death of a baby**		
Yes	40	6.5
No	578	93.5
**Any of children hospitalized**		
Yes	104	16.8
No	514	83.2
**Stressful life event during pregnancy**		
Yes	21	3.4
No	597	96.6

**Table 3:** Social support among postpartum women’s, from health centers of four sub-cities of Addis Ababa city Administration, Ethiopia, (N= 618).

Characteristics	Frequency	Percent
**Abuse/domestic violence **		
Yes	87	14.1
No	531	85.9
**Satisfy with marriage **		
Yes	505	81.7
No	113	18.8
**Husband support **		
Yes	514	83.2
No	104	16.8
**Relatives present during labor**		
Yes	560	90.6
No	58	9.4
**Satisfy in relation with mother-in-law**		
Yes	440	71.2
No	178	28.8

**Table 4:** EPDS (Edinburgh postnatal depression scale) responses among postpartum women’s, from health centers of four sub-cities of Addis Ababa city Administration, Ethiopia, (N= 618).

**Characteristics **	**Frequency**	** Percent**
**Experienced laugh and see funny side of things **		
As much as always I could	394	63.8
Not quite so much now	102	16.5
Definitely not so much now	53	8.6
Not at all	69	11.2
**Look forward with enjoyment to things **		
As much as I ever did	385	62.3
Rather less than I used to	125	20.2
Definitely less than I used to	60	9.7
Hardly at all	48	7.8
**Blamed yourself unnecessarily **		
No never	309	50.0
Not very often	119	19.3
Yes some of the time	132	21.4
Yes most of the time	58	9.4
**Been anxious or worried for no good reason **		
No not at all	319	51.6
Hardly ever	70	11.2
Yes sometimes	175	28.3
Yes very often	54	8.7
**Felt scared or panic for no good reason **		
No not at all	375	60.7
No, not much	102	16.5
Yes, sometimes	111	18.0
Yes, quite a lot	30	4.9
**Things have been on top of you **		
No I have been coping	388	62.8
No most of the time	166	18.8
Yes sometimes I haven’t been coping as well as usual	89	14.4
Yes most of the time I haven’t been able to cope at all	25	4.0
**Difficult to sleep **		
No, not at all	375	60.7
Not, very often	144	23.3
Yes sometimes	73	11.8
Yes most of the time	26	4.2
**Felt sad or miserable**		
No, not at all	386	62.5
Not, very often	149	24.1
Yes, quite often	62	10.0
Yes, most of the time	21	3.4
**So unhappy you have been crying **		
No, never	410	63.3
Only occasionally	149	24.1
Yes quite often	41	6.6
Yes, most of the time	18	2.9
**Thought of harming your self **		
Never	544	88.0
Hardly ever	41	6.6
Sometimes	31	5.0
Yes, quite often	2	0.3

**Table 5:** Bivariate and multivariate logistic regression analysis of postpartum depression (N=618).

**Variables **	**Depressed n(%)**	**Not Depressed** **n(%)**	**COR(95% C.I)**	**AOR(5% C I).**
Age				
15-24	35(25.4)	103(74.6)	1.06(0.54,2.07)	0.99(0.42,2.36)
25-34	92(22.4)	318(77.6)	0.90(0.50,1.63)	1.22(0.58,2.57)
>=35	17(24.3)	53(75.7)	1	1
Educational status				
No formal education	44(37.9)	72(62.1)	2.77(1.76,4.38)*	1.52(0.77,2.99)
Primary education	31(25.8)	89(74.2)	1.58(0.97,2.57)	1.29(0.68,2.44)
Secondary and above	69(18.1)	313(81.9)	1	1
Marital status				
Married	105(18.4)	466(81.6)	**1**	1
Unmarried	39(83.0)	8(17.0)	21.64(9.82,47.65)*	7.70(3.03,19.54)*
Occupation				
Employed	67(19.9)	270(80.1)	0.66(0.45,0.96)	0.99(0.58,1.70)
Unemployed	77(27.4)	204(72.6)	1	1)
History of Children death				
Yes	21(52.5)	19(47.5)	4.1(2.13,7.85)*	2.86(1.28,6.38)*
No	123(21.3)	455(78.7)	1	1
Relatives present during labor				
Yes	102(18.2)	458(81.8)	1	1
No	42(72.4)	16(27.6)	11.80(6.4,21.80)*	**5.47(2.58,11.58**)*
Planed pregnancy				
Yes	51(11.6)	388(88.4)	0.12(0.08, 0.18)*	0.20(0.12,0.33)*
No	93(52.0)	86(48.0)	1	1
History of Substance use				
Yes	31(34.1)	60(65.9)	1.89 (1.17,3.06)*	2.57(1.38,4.81)*
No	113(21.4)	414(78.6)	1	1
History of abortion				
Yes	29(29.0)	71(71.0)	1.43(0.89,2.31)	0.78(0.43,1.44)
No	115(22.2)	403(77.8)	1	1
Desired sex of infant				
Undesired	30(22.1)	106(77.9)	0.71(0.44,1.16)	0.70(0.37,1.31)
Desired	36(17.4)	171(82.6)	0.53(0.34-0.83)	0.79(0.44,1.44)
I don’t mind	78(28.4)	197(71.6)	1	1
Income Difficulty				
Yes	90(42.9)	120(57.1)	4.92(3.31,7.30)*	2.68(1.63,4.42)*
No	54(13.2)	354(86.8)	1	1

*p <0.01,
